# Editorial: Diagnosis and identification of novel disorders and ultra-rare disorders in science and clinical routine

**DOI:** 10.3389/fgene.2024.1522931

**Published:** 2024-11-20

**Authors:** Hacer Durmus

**Affiliations:** Department of Neurology, Istanbul Faculty of Medicine, Istanbul, Türkiye

**Keywords:** genetic, clinical care, critically ill, rapid exome sequencing, exom squencing

The application of rapid exome sequencing (rES) has emerged as a crucial advance in diagnostic landscape, especially for critically ill patients presenting with rare diseases. This editorial reflects findings from a comprehensive study involving 575 patients that highlighted the transformative impact of rES on clinical decision-making and patient outcomes (Marouane et al.).

In recent years, rES has become the preferred genetic testing modality for critically ill patients, including neonates and young infants, in urgent clinical situations (Ouyang et al., 2021; McDermott et al., 2022; Wells et al., 2022). Its ability to provide timely diagnoses can significantly guide management decisions and improve clinical care pathways. The study, conducted over 4 years (2016–2019) provides valuable insights into the operational effectiveness and clinical utility of rES. The study reported a notable increase in rES referrals, escalating from two in the first quarter of 2016 to ten per week by late 2019. This increase reflects growing recognition of rES as a critical tool in diagnosing complex genetic disorders. The median turnaround time for results improved from 17 days to 11 days, this highlighted advances in sequencing technology and laboratory efficiencies (Marouane et al.).

The overall diagnostic yield was 30.4%, with variations observed across different clinical entities. For instance, craniofacial anomalies showed a high diagnostic yield of 58.3%, whereas conditions like severe combined immune deficiency yielded no diagnoses at all. These findings suggest that rES, although not universally effective for all conditions, is vital for many patients and offers information that can change clinical management even in the absence of a definitive diagnosis (Marouane et al.).

The importance of rES extends beyond providing definitive genetic diagnoses. Even if genetic causes remain elusive, information gleaned from rES may influence clinical decisions such as direction of treatment or the need for further investigation. This dual effect in both making diagnoses and informing clinical strategies highlights the multifaceted role of rES in patient care.

Implementation of rES should be accompanied by careful ethical considerations, particularly regarding informed consent and genetic counselling. In high-stake situations, it is very important to ensure that patients and families understand the implications of genetic tests (Cakici et al., 2020). Clinicians must navigate the complexity of informing uncertain or negative findings while remaining sensitive to the emotional impact on families.

As rES continues to evolve, its integration into routine clinical practice for older patients should be prioritized. The potential for RES to guide treatment decisions in cancer care and other adult-onset conditions is significant and requires further investigation. Ongoing research should focus on optimizing diagnostic strategies and understanding the broader implications of genetic findings in diverse cohorts.

Lessons learned from the use of rapid exome sequencing in critically ill patients underline its fundamental role in modern medicine era. The ability to quickly identify genetic causes of rare diseases not only increases diagnostic accuracy but also improves clinical outcomes. As these technologies continue to be developed and their applications expanded, the hope is to further close the gap between genetics and clinical practice, ultimately benefiting patients across the healthcare.
